# Effects of the Immobilization of the Upper Extremities on Spatiotemporal Gait Parameters during Walking in Stroke Patients: A Preliminary Study

**DOI:** 10.1155/2020/6157231

**Published:** 2020-06-02

**Authors:** Seung-hyeon Hong, So-young Jung, Hyeon-kyung Oh, So-hyeon Lee, Young-keun Woo

**Affiliations:** Department of Physical Therapy, College of Medical Sciences, Jeonju University, Jeonju, Republic of Korea

## Abstract

**Background:**

The purpose of this study was to investigate the effects of upper extremity immobilization and consequent walking speed on spatiotemporal gait parameters in stroke patients with hemiparesis.

**Methods:**

The following variables were assessed or measured in 29 stroke patients: age, height, weight, disease duration, Korean version of the Mini-Mental State Examination (MMSE-K), Berg balance scale (BBS-K), functional gait assessment (FGA-K), cause of the disease (type of lesion), and hemiparetic side. The measurement of gait was performed using two pressure plates of 1.5 m to create a 3 m walking distance and leaving 1.5 m of extension at both start and end, to ultimately create a 6 m walking distance that the patient could walk through. The following gait patterns were randomly selected based on card draws: self-selected walk speed (SW), self-selected walk speed with immobilized upper extremities (SWI), fast walking (FW), and fast walking with immobilized upper extremities (FWI). Each patient was assessed for four different gait patterns, with three measurements per pattern (12 gait measurements in total).

**Results:**

While there were significant differences in the stride length, step width, velocity, and step length of the paretic side between self-selected walk speed (SW) and SWI, FWI did not show significant changes in any of the tested parameters.

**Conclusions:**

Immobilization of the upper extremities may affect walking at self-selected walk speeds. A comprehensive training program including upper extremity movement should be established for gait rehabilitation. *Clinical Trial Registration*. This trial is registered at http://cris.nih.go.kr/cris.

## 1. Introduction

Stroke is accompanied by multiple functional impairments, including impairment of motor, cognitive, and sensory function, caused by damage to the blood vessels in the brain. More specifically, hemiparesis—partial paralysis of one side of the body—is the classical symptom of stroke [1]. Hemiparetic symptoms lead to an increased body weight on one side of the body while walking, resulting in further decrease in the weight-bearing capacity of the paretic side secondary to motor weakness, asymmetrical muscular tone, sensory loss, perceptual deficits, and consequently, an abnormal gait pattern [2–4]. For these reasons, improving gait function is one of the most important aspects of functional rehabilitation in stroke patients with hemiparesis [5].

Walking is the most fundamental process involved in performing daily activities, and typical walking involves coordination of several joints in both upper and lower extremities to create movement. The role of the upper extremities in walking is to rotate the upper extremities in the opposite direction to prevent compensation from rotation of the pelvis in the same direction as walking and consequently to maintain bodily balance [6]. The upper extremities play a role in trunk stabilization, preventing excessive use of energy [6]. Upper extremity movement plays a crucial role in achieving high walking velocity by becoming active, after being passive, with an increase in walking speed from slow to normal [7]. Generally, restricting upper extremity movement during walking results in slower and less stable walking [8]. Consequently, upper extremity immobilization during walking can induce negative changes in gait characteristics and movement-control patterns [9].

Brooke et al. [10] showed that preventing symmetrical, pendulum-like movements of the upper extremities caused difficulties in walking. Faghri et al. [11] found that the use of upper extremity slings in poststroke hemiparesis patients to reduce subluxation or pain not only increased flexor synergy of the extremities but also hindered functional mobility. Yavuzer and Ergin [12] compared a group of patients with hemiparesis and healthy controls, using an upper extremity sling to cause upper extremity immobilization, and found a reduced speed of gait during training sessions.

These previous studies reported changes in parameters such as walking speed caused by immobilization of the upper extremities during walking. Kawajiri et al. [13] suggested that the maximum walking speed could be a prognostic factor in patients with mild strokes. Grau-Pellicer et al. [14] reported that the walking speed is an important factor for regaining independent outdoor ambulation in community mobility. Hwang and Yoon [15] reported the effects of sling immobilization of the affected extremity on muscle activity and kinematic data of lower extremity rather than clinical walking parameters such as speed. Taken together, these findings suggest that the effects of upper extremity immobilization and consequent walking speed on spatiotemporal gait parameters in stroke patients with hemiparesis have not been reported. Therefore, we tested the positive or negative effects of upper extremity immobilization on self-selected walk speed, fast walking speeds, and spatiotemporal gait parameters in stroke patients with hemiparesis.

## 2. Methods

### 2.1. Subjects

The subjects of this study were 29 stroke patients with hemiparesis, admitted to a rehabilitation center in Jeonju, Korea. All subjects understood the purpose of this study and agreed to participate prior to study initiation, and informed consent was obtained from all patients prior to participation. This study was approved by the institutional review board of Jeonju University (JJIRB-180712-HR-2019-0412). The subjects' ages, heights, weights, disease durations, Korean version of the mini-mental state examination (MMSE-K), Berg balance scales (BBS-K), functional gait assessments (FGA-K), causes of the disease (type of lesion), and hemiparetic sides were assessed or measured. Inclusion criteria were as follows: patients initially diagnosed with stroke by a physician, chronic stroke patients with persistent hemiparesis for over 6 months after the stoke onset, and patients who have understood the directions for performing the MMSE-K provided by the investigator. The following were exclusion criteria: patients who had orthopedic surgery in either upper or lower extremities within the past year, mental disorders (MMSE − K ≤ 17), patients who taking medications for balance control such as neurotropic drugs during the study period, and patients with ankle-foot orthosis (AFO) to prevent excessive foot dragging (Table 1).

### 2.2. Measurement Tools and Methods

#### 2.2.1. BBS-K

BBS-K is a scale that evaluates both static and dynamic balance abilities. It consists of 14 tasks, with a 5-point ordinal scale ranging from 0 (lowest level of function) to 4 points (highest level of function), with a maximum overall score of 56. Each task can be divided into the following categories: seated tasks such as sitting upright on a chair without leaning on the back of a chair; standing tasks such as standing still without holding onto anything, standing still with eyes closed and without holding onto anything, looking left and right, picking up items on the floor, standing with one foot perpendicular to the other foot, and putting arms out in front while standing; and posture changing tasks such as standing up from a seated position, sitting down from a standing position, moving from a chair to another chair, rotating 360° in the same spot, and stepping onto a footrest of fixed height in walking motion (alternating between two feet). This tool is the gold standard, with high reliability, as demonstrated by an intraobserver reliability of ICC = 0.99 and interobserver reliability of ICC = 0.98 [16]. The Korean version of BBS showed interrater reliability of 0.97 and intrarater reliability of 0.95 [17].

#### 2.2.2. MMSE-K

MMSE-K was developed to evaluate the cognitive function. There are six evaluation criteria: orientation, memory registration, memory recall, attention and calculation, linguistic ability, and comprehension and judgment. Patients can provide either verbal responses, written responses, or drawings depending on items. The total score is 30, with a score of ≥24 considered as normal, 18–23 as mild cognitive disorder, and ≤17 as severe cognitive disorder [18]. The interobserver reliability of this tool is ICC = 0.99 [19].

#### 2.2.3. FGA-K

FGA-K was developed to evaluate gait in elderly individuals with a high risk of falling. Overall, the following ten tasks are included in this assessment: flat-surface walking, changing walking velocity, rotating the head sideways while walking, moving the head up and down while walking, rotating using one foot as an axis while walking, walking over an obstacle, walking on a narrow surface, walking with eyes closed, walking backward, and climbing the stairs. Each task is evaluated using a 4-point scale based on the severity: 0 for the most severe form of dysfunction, 1 for intermediate dysfunction, 2 for mild dysfunction, and 3 for no signs of dysfunction. Test-retest reliability (repeatability) of the tool when used in a stroke patient is ICC = 0.97, and interobserver reliability is ICC = 0.94 [20]. The Korean version of FGA showed an interrater reliability of 0.91 and intrarater reliability was 0.92 [21].

#### 2.2.4. Upper Extremity Sling

**Figure 1:**
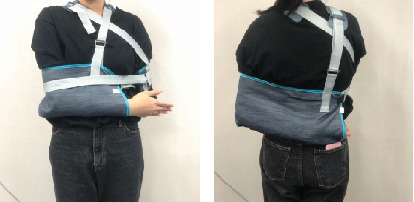
Anterior and posterior views of immobilization of the upper extremities.

An upper extremity sling (Wooa Co., Jeonju, Korea) was used to immobilize the patient's upper extremities. The affected side was immobilized in an anterior direction, and the unaffected side was immobilized in a posterior direction. To ensure that the upper extremities are not moving, the patients' upper extremities were attached to the trunk using a knot (Figure 1).

#### 2.2.5. Measurement of Spatiotemporal Gait Parameters While Walking

A pressure-based gait analyzer FDM (Zebris Medical GmbH, Germany) was utilized to measure the spatiotemporal gait parameters while walking. FDM measures up to 120 Hz/sec and 1580 mm × 605 mm × 21 mm and contains 11,264 built-in pressure sensors. The measurement of gait was performed by connecting two 1.5 m pressure plates to create a 3 m walking distance and leaving 1.5 m of extension at both start and end, to ultimately create a 6 m walking distance that the patient walks through. The gait parameters, such as stride length, step width, double-stance phase, stride time, cadence, velocity, step length, percentage of stance phase, loading response, midstance, preswing, swing phase, and step time, were automatically calculated by Zebris FDM Software Suite. The investigator provided each patient with an explanation on the experimental process, and patients performed 12 gaits in total (six times without immobilized upper extremities and six times with immobilized upper extremities, three with a self-selected walk speed, and three with a fast walk speed). The following gait patterns were randomly selected based on card draws: self-selected walk speed (SW), self-selected walk speed with immobilized upper extremities (SWI), fast walking (FW), and fast walking with immobilized upper extremities (FWI). All measurements were made while the patient was walking with comfortable clothes that did not hinder walking. After the patients had fully understood the experimental process, they were given the following instructions for each of the gait patterns: “Please walk as you would walk with a self-selected pace” for self-selected walk speed and “Please walk as if you need to catch the bus right in front of you, but making sure you do not fall” for fast walking. A 1-minute rest time was given between gaits of different patterns. Each patient was assessed for four different gait patterns, with three measurements per pattern (12 gait measurements in total). The average of triplicate measurements was used as the final data value. In addition, two therapists accompanied each patient side by side while they were walking to ensure patient safety.

### 2.3. Statistical Methods

Analysis of data in this study was performed using SPSS for Windows version 25.0 (IBM Inc., Chicago, IL, USA). General characteristics and clinical assessment indices were analyzed using descriptive statistics and frequency analyses. To compare the differences based on the four gait patterns, repeated one-way ANOVA was utilized. The Bonferroni post hoc test was used to compare the differences among the gait patterns. For all outcomes, the statistical significance was set at *α* = 0.05.

## 3. Results

### 3.1. Comparison of Spatiotemporal Gait Parameters While Walking with Upper Extremity Immobilization

Differences in spatiotemporal gait parameters while walking with upper extremity immobilization are shown in Table 2. Between SW and SWI, there were significant differences in stride length, step width, and velocity (*p* < 0.05). Meanwhile, between FW and FWI, there was no significant difference in any of the tested parameters (*p* > 0.05).

### 3.2. Comparison of Spatiotemporal Gait Parameters on the Paretic Side with Upper Extremity Immobilization

Differences in spatiotemporal gait parameters on the paretic side with upper extremity immobilization are displayed in Table 3. Between SW and SWI, step length was the only parameter showing a significant difference (*p* < 0.05). Between FW and FWI, there was no significant difference in any of the tested parameters (*p* > 0.05).

## 4. Discussion

We assessed the effect of upper extremity immobilization on the gait of stroke patients. The subjects were asked to perform SW and FW with and without immobilization of the upper extremities to assess the differences in spatiotemporal gait parameters. We found that while there were significant differences in the stride length, step width, velocity, and step length of the paretic side between SW and SWI, there was no significant difference in any of the tested parameters between FW and FWI.

The stride length decreased from 79.13 ± 22.86 cm during SW to 76.07 ± 22.48 cm during SWI, whereas the step width increased from 18.64 ± 3.49 cm during SW to 19.59 ± 2.95 cm during SWI. Moreover, velocity decreased from 62.27 ± 26.57 cm/s during SW to 57.77 ± 25.57 cm/s during SWI. However, there was no significant difference in any of these parameters between FW and FWI. Wagenaar and van Emmerik [7] found that, in general, an increase in walking velocity results in changes in upper extremity movement from passive to active and aggressive. Eke-Okoro et al. [22], in their study on healthy adults, demonstrated that the immobilization of both upper extremities results in a significantly greater reduction in walking velocity than that obtained when upper extremities are not immobilized, and the immobilization of one upper extremity causes a significantly greater reduction in walking velocity and stride length than that obtained when upper extremities are not immobilized. Morrey et al. [23] suggested that elbow damage restricts upper extremity movement and consequently affects walking function. Ford et al. [9] assessed healthy adult subjects, with immobilization of either the dominant or nondominant side, and found a greater reduction in rotation of the pelvis, chest, and trunk than obtained during a state of nonimmobilization. These authors mentioned that when there is no restriction in the movement of upper extremities, cooperativity between the upper and lower extremities improves, consequently increasing gait velocity. Williams et al. [24] demonstrated that the location of upper extremities, fixed by equipment (i.e., arm sling), hindered functional activity of the upper extremities. Trehan et al. [25] reported reductions in gait velocity and stride length when the subjects' elbows were fixed at 30°, 90°, or 120°, and they were equipped with a 90° fixed orthosis during walking. In agreement with these findings, the outcomes in our study demonstrated reduced stride length, step length, gait velocity, and increased step width during SW compared to SWI. These outcomes are likely due to an inherent mechanism that ensures safer balance in stroke patients, under the condition of immobilized upper extremities. Furthermore, the step length of the paretic side was reduced from 39.61 ± 11.33 cm to 37.47 ± 12.03 cm, likely due to compensation in order to ensure better balance of the body.

We found no significant difference in any of the parameters between FW and FWI. Eke-Okoro et al. [22], in their study on normal subjects, demonstrated that walking at the fastest velocity with immobilized upper extremities resulted in a significant reduction in gait velocity. Potempa et al. [26] showed that upper extremity movement had a positive effect on general walking, as a first step in gait safety. Generally, immobilization of the upper extremities indirectly induced limited upper extremities with the trunk that led to preventing rotational movement of trunk during walking. However, in our study, the subjects were stroke patients with hemiparesis, and this is the likely the reason for the different outcomes. When the subjects were asked to perform FW, most of parameters were similar between FW and FWI; we hypothesized that the speed of gait was relatively slowed in fast walking conditions, despite of requirement for fast walking. Another possibility is that, in previous studies, stroke patients needed to organize factors other than swinging upper extremities for increasing speed. Jonkers et al. [27] reported that impaired power generation of the ankle and hip led to potential limited walking speed in stroke patients. Franceschin et al. [28] reported that asymmetry between affected and unaffected sides in hemiparesis due to stroke reduced gait ability. Knikou and Rymer [29] observed that stroke patients persistently required visual and proprioceptive information for other dynamic balance-related factors and balance manipulation, rather than the movement of upper extremities during walking. In individuals without neurological damage, cooperative patterns between upper and lower extremities rely on the walking velocity, and upper extremities exhibit minimal movement at a relatively slow gait pattern [30].

According to these results, there is a need to consider movement of upper extremities during slow or self-selected walking in addition to fast walking. Kawashima et al. [31] and Stephenson et al. [32] suggested a comprehensive walking rehabilitation program, involving upper extremity movement in patients with spinal cord injury, which would induce cooperative movement of the legs during walking via cooperative activity between the upper and lower trunk. Therefore, alternating upper extremity movement would need to be self-selected during walking training for stroke rehabilitation; however, under conditions of fast walking, there is the need to consider other factors for improving walking speed in stroke rehabilitation.

We used slings for immobilization the affected upper extremity in the anterior direction and the unaffected upper extremity in the posterior direction during walking. Most of the affected upper extremities of stroke patients were limited in terms of range of motion at the shoulder joints; therefore, we used immobilization of the affected side in the anterior position. Siragy et al. [33] reported that active arm swing led to trunk linear and angular velocity, as well as the center of mass movement during walking. Therefore, we tried to prevent compensatory motion of the affected upper extremity using the unaffected upper extremity in case of both upper extremities with same directional immobilization, and we also tried to make a similar distribution of center of mass in the upper extremities during walking.

This study have a few limitations. First, we did not include BBS-K and FGA-K to analyze the effect of immobilization of upper extremities during walking or upper extremity ability and walking ability of stroke patients during various walking conditions. Moreover, participants in this study were found to have varying FGA-K scores, which may have correlated with gait outcomes. Nevertheless, patients were not divided into groups based on FGA-K scores because the sample size was too small and the analyses would be complex. Second, limited spatiotemporal gait parameters were utilized for the analysis, and analyses of other epidemiological data such as MMSE-K from events that occur during walking were not performed. Finally, other physical functions that can affect fast walking should also be analyzed. Therefore, further studies investigating the effect of upper extremity immobilization based on upper extremity ability, walking ability, and balance are required.

## 5. Conclusion

While there were significant differences in the stride length, step width, velocity, and step length of the paretic side between SW and SWI, immobilization of the upper extremities during FW did not induce statistically significant changes in any of the tested parameters. Because immobilization of the upper extremities may affect walking at a self-selected speed, a comprehensive training program for self-selected walk speed including upper extremity movement rather than immobilization or uninvolved movement of the upper extremity during gait rehabilitation is not appropriate for gait rehabilitation. Other factors that can affect FW rehabilitation, aside from upper extremity movement, should be analyzed in the future studies.

## Figures and Tables

**Table 1:** General characteristics of participants.

Parameters	Mean ± standard deviation
Age (years)	60.69 ± 12.20
Height (cm)	166.46 ± 8.37
Weight (kg)	66.88 ± 10.14
Duration	12.95 ± 4.96
MMSE	24.14 ± 3.51
BBS	40.83 ± 10.53
FGA	17.69 ± 5.44
Sex (male/female)	22/7
Type of lesion (hemorrhagic/infarction)	17/12
Hemiplegic side(left/right)	18/11

Abbreviations: MMSE: mini-mental state examination; BBS: Berg balance scale; FGA: functional gait assessment.

**Table 2:** Comparison of spatiotemporal gait parameters while walking based on upper extremity immobilization.

Parameters	SW^a^	SWI^b^	FW^c^	FWI^d^	F	*p* value	Post hoc test^e^ (*p* value)
Stride length (cm)	79.13 ± 22.86	76.07 ± 22.48	89.96 ± 29.93	87.03 ± 29.13	18.886	0.001	1 (0.040) 2 (0.001) 3 (0.037) 4 (0.001) 5 (0.001)

Step width (cm)	18.64 ± 3.49	19.59 ± 2.95	18.71 ± 3.34	19.21 ± 3.35	5.625	0.001	1 (0.006) 4 (0.028)

Double stance phase (%)	40.28 ± 9.76	42.41 ± 12.37	37.35 ± 12.30	37.68 ± 12.54	13.239	0.001	2 (0.017) 4 (0.001) 5 (0.001)

Stride time (sec)	1.36 ± 0.29	1.44 ± 0.38	1.20 ± 0.35	1.26 ± 0.39	18.175	0.001	2 (0.001) 4 (0.001) 5 (0.003)

Cadence (steps/min)	91.63 ± 17.19	88.23 ± 17.85	106.97 ± 23.51	103.81 ± 24.47	9.511	0.001	4 (0.001) 5 (0.001)

Velocity (cm/s)	62.27 ± 26.57	57.77 ± 25.57	83.92 ± 40.68	78.86 ± 40.90	15.134	0.001	1 (0.009) 2 (0.001) 3 (0.010) 4 (0.001) 5 (0.001)

^a^Self-selected walk speed. ^b^Self-selected walk speed with immobilization of upper extremities. ^c^Fast walking. ^d^Fast walking with immobilization of upper extremities. ^e^Number indicates the following results from post hoc tests (*p* value): 1 = significant difference between SW and SWI at *p* < 0.05; 2 = significant difference between SW and FW at *p* < 0.05; 3 = significant difference between SW and FWI at *p* < 0.05; 4 = significant difference between SWI and FW at *p* < 0.05; 5 = significant difference between SWI and FWI at *p* < 0.05; 6 = significant difference between FW and FWI at *p* < 0.05.

**Table 3:** Comparison of spatiotemporal gait parameters on the paralyzed side based on upper extremity immobilization.

Parameters	SW^a^	SWI^b^	FW^c^	FWI^d^	F	*p* value	Post hoc test^e^ (*p* value)
Step length (cm)	39.61 ± 11.33	37.47 ± 12.03	44.59 ± 15.94	43.14 ± 15.74	18.119	0.001	1 (0.004) 2 (0.002) 4 (0.001) 5 (0.001)

Stance phase (%)	69.66 ± 6.81	70.11 ± 6.24	67.61 ± 6.21	67.74 ± 6.59	2.591	0.105	4 (0.001) 5 (0.003)

Loading response (%)	22.21 ± 4.98	23.53 ± 6.89	20.80 ± 6.97	20.83 ± 7.09	6.195	0.001	4 (0.002) 5 (0.036)

Mid stance (%)	26.68 ± 7.37	26.26 ± 7.78	28.31 ± 7.37	28.25 ± 7.32	5.440	0.002	4 (0.001) 5 (0.042)

Pre-swing (%)	19.71 ± 5.22	20.75 ± 6.11	18.38 ± 6.51	18.64 ± 6.40	8.167	0.001	4 (0.001) 5 (0.001)

Swing phase (%)	31.45 ± 5.19	29.89 ± 6.24	32.39 ± 6.21	32.26 ± 6.59	7.077	0.001	4 (0.001) 5 (0.003)

Step time (sec)	0.70 ± 0.18	0.73 ± 0.22	0.61 ± 0.21	0.64 ± 0.22	19.814	0.001	2 (0.001) 4 (0.001) 5 (0.001)

^a^Self-selected walk speed. ^b^Self-selected walk speed with immobilization of upper extremities. ^c^Fast walking. ^d^Fast walking with immobilization of upper extremities. ^e^Number indicates the following results from post hoc tests (*p* value): 1 = significant difference between SW and SWI at *p* < 0.05; 2 = significant difference between SW and FW at *p* < 0.05; 3 = significant difference between SW and FWI at *p* < 0.05; 4 = significant difference between SWI and FW at *p* < 0.05; 5 = significant difference between SWI and FWI at *p* < 0.05; 6 = significant difference between FW and FWI at *p* < 0.05.

## Data Availability

The data that supports the findings of this study are available upon request from the corresponding author. The data are not publicly available due to restrictions (e.g., containing information that could compromise the privacy of research participants)..

## Additional Points

The Clinical Research Information Service Number is KCT0004057, and the final confirmation is on 12, June 2019, retrospectively registered http://cris.nih.go.kr/cris.
